# Urinary ascites secondary to delayed diagnosis of laparoscopic bladder injury

**DOI:** 10.4103/0972-9941.65165

**Published:** 2010

**Authors:** Hazem Al-Mandeel, Abeer Qassem

**Affiliations:** Department of Obstetrics and Gynecology, King Khalid University Hospital, King Saud University, Riyadh, Saudi Arabia

**Keywords:** Inadvertent bladder injury, laparoscopy, urinary ascites

## Abstract

We present a case of urinary ascites in a young woman secondary to unrecognized bladder injury during gynaecologic laparoscopic surgery. Delayed diagnosis occurred due to the absence of expected changes in serum biochemistry, which made the diagnosis of urinoma less likely. High suspicion of bladder injury following laparoscopic surgery should be present in patients with ill-defined symptoms even if no biochemical changes are seen. The case demonstrates important points in relation to the consequences of delayed diagnosis as well as overview on detection and prevention of such injury.

## INTRODUCTION

Urinary bladder injury is a recognized complication of laparoscopic surgery, and it is the most common organ injured during gynecological procedures.[1] However, when it occurs, it is usually recognized intra-operatively and it is either repaired or treated conservatively with continuous drainage of the bladder for 5 to 7 days.

Herein we present a case of inadvertent and unrecognized cystotomy during laparoscopic salpingo-oophorectomy that led to post-operative urinary ascites and other consequences.

## CASE REPORT

A 19-year-old female, not known to have any past medical or surgical history, underwent urgent operative laparoscopy for acute abdomen and ultrasound finding of a large ovarian cyst. At the beginning of the procedure, a 14-French Foley's catheter was left in the bladder for continuous drainage. For laparoscopic access, Veress needle was introduced into the peritoneal cavity through an infraumbilical skin incision. Following pneumoperitoneum, 11-mm disposable plastic trocar was used. Three ancillary trocars were inserted under direct visualization. One trocar through a suprapubic incision (11 mm) and two in each of the lower quadrants of the abdomen (size: 5mm each). Large left dermoid cyst about 12 × 10 cm with ovarian torsion was found. Left oophorectomy with the use of electrosurgical energy was done. The cyst was accidently punctured and appeared to be a dermoid cyst, which was removed by endo-bag followed by copious irrigation. On the second postoperative day, the patient started to complain of voiding difficulty and passing small volumes of urine and she was treated with hydration and indwelling catheter for 24 h.

On postoperative day 3, she developed fever and abdominal distension and was not passing any flatus. On examination, bowel sounds were found to be absent. At that time urine culture was positive for *Klebsiella pneumoniae* and parenteral antibiotic was started. Abdominal X-ray showed dilated bowel loops with multiple air fluid levels. Her haemoglobin was 11.7, platelets 263, white blood cells 8.1, urea 3.4, creatinine 94, potassium 3.5 and sodium level of 141. Postoperative illeus was diagnosed and she was kept fasting with good hydration.

On post operative day 4, abdominal and pelvic ultrasounds were done for her that showed free fluid in the upper abdominal quadrants, and multiseptated partially contained fluid at right side of the lower abdomen area measuring 14.5 × 4.5 cm, which thought to be part of the fluid used for irrigation. Two days later, the patient developed diarrhoea, and repeated laboratory tests were normal except for leucocytosis (16.8) and hypokalaemia (3.1).

Intestinal injury was suspected and contrast-enhanced CT scan revealed moderate to severe ascites, and no features to suggest abscess or bowel perforation. Aspiration, under US guidance, of straw-coloured ascetic fluid was performed resulting in slight improvement of the abdominal distension and the patient's condition.

Biochemical analysis of the aspirated liquid revealed elevated urea (36.3), creatinine (1551) and potassium (7.9). Culture of the fluid was positive for *Klebsiella pneumonia*. Urologist performed retrograde cystouretrogram, which confirmed an extravasation of the contrast from the bladder. Cystoscopy showed bladder injury (3 cm size cystotomy) to be present at the dome of the bladder; both ureters were patent and intact.

The patient underwent urgent laparotomy, and the cystotomy was repaired in two layers. The bowel was noted to be distended and inflamed and multiple small collections of urinomas between matted bowel loops were evacuated by blunt dissection and irrigation. Postoperatively, the patient continued to have absent bowel sounds for 1 week followed by diarrhoea which settled in few days. Spikes of fever continued for almost two weeks following the laparotomy despite parenteral antibiotic treatment. Cysotogram was repeated and showed normal integrity of the bladder.

The patient was discharged home in good condition and her 2 months' follow-up revealed complete recovery.

## DISCUSSION

In a 1998 review, the incidence of bladder injury during laparoscopic procedures ranged from 0.02% to 8.3%.[1] It was more frequent in gynaecologic procedures, and intraoperative diagnosis was made only in 53% of cases.[1] Most publications indicate that individuals who have abnormal pelvic anatomy because of adhesions from previous surgery or intra-abdominal disease such as endometriosis or pelvic inflammatory disease are more vulnerable to such injury. However, such injuries have been reported even after simple diagnostic laparoscopy without risk factors.[2]

In one report, electrosurgical dissection was found to be the most likely cause of injury, followed by blunt dissection of the bladder followed by trocar insertion.[3] Others reported that half of all complications are thought to occur at insertion of the trocar, and the urinary bladder is especially at risk because of its location, distensibility and thin wall.[2] This was the most likely cause of cystotomy in this case.

Signs indicating bladder injury intraoveratively are bloody urine and the presence of gaseous distention of the urine bag indicative of a communication between the bladder and the intraperitoneal space (the catheter bag sign).[4] The injury may be confirmed by instillation of methylene blue in the bladder, but bladder perforations are not always easy to detect during laparoscopy especially in the absence of above signs.[4]

If bladder injury is missed intraoperatively, the patient may present with voiding difficulty, oliguria, haematuria, pyrexia, abdominal distension, nausea and vomiting and peritonitis secondary to the formation of urinoma as early as 1 to 2 days after the incident.[5] Fever may occur, which makes it even more difficult to distinguish from urinary tract infection as happened in our case.[6] Even though oliguria was noted in our patient during the early postoperative period, there was no other clear evidence to raise our suspicion.

In most patients with urinary ascites, there may be great disturbances in serum biochemistry mimicking renal failure such as elevation in the urea and creatinine as well as hyperkalaemia and hyponatraemia. This occurs because urine has higher concentration of urea, creatinine and potassium than serum resulting in diffusion of these substances into the lower concentration gradient, while sodium diffuses in the opposite direction. In our patient the serum urea, creatinine and potassium were not raised to an abnormal level as previously reported possibly because the volume of urinary ascites was not too large with good renal function.[7,8] No doubt, the absence of the biochemical derangements added to delayed diagnosis of urinoma.

In cases of significant accumulation of urine in the peritoneal cavity, the patients may complain of respiratory distress from pleural effusion resulting from shift of urine from the peritoneal cavity into the pleural cavity through the diaphragm and from severe ascites caused by the urinary collection in the peritoneal cavity which causes irritation to the diaphragm.[9]

When bladder injury is suspected, abdominal ultrasound will determine the presence or absence of ascites.[7] The site of injury is frequently defined by conventional retrograde cystography with an accuracy of 85% to 100%. False negative findings occur when the bladder perforation is small and the bladder is not distended adequately. CT cystogram has the ability to show even small perforation in addition to detecting additional abdominal or pelvic pathologies.[8] If the bladder injury is recognized intraoperatively, it should be repaired surgically. When the injury is less than 1 cm it can be treated by continuous drainage with Foley's catheter for 5-7 days.[6]

Prevention of bladder injury depends on knowledge of anatomy, emptying the bladder before starting the procedure preferably with indwelling Foley's catheter, particularly if bladder dissection is required or when a long operation is expected.[10] In addition, secondary trocars should always be placed under direct vision and sufficiently above the symphysis to avoid bladder perforation.

In the presence of adhesions, the bladder may be insufflated with 200 to 250 ml of gas or liquid to identify the muscularis margin of the bladder and allow accurate sharp dissection.[11]

## CONCLUSION

The diagnosis of inadvertent bladder injury may be delayed due to the insidious development of non-specific symptoms. High suspicion of bladder injury following laparoscopic surgery should be present in patients with ill-defined symptoms even if no biochemical changes are seen.
